# Genetically Based Physiological Responses to Overwinter Starvation in Gibel Carp (*Carassius gibelio*)

**DOI:** 10.3389/fendo.2020.578777

**Published:** 2020-11-19

**Authors:** Wenjie Xu, Hongyan Li, Liyun Wu, Bo Dong, Junyan Jin, Dong Han, Xiaoming Zhu, Yunxia Yang, Haokun Liu, Shouqi Xie

**Affiliations:** ^1^ State Key Laboratory of Freshwater Ecology and Biotechnology, Institute of Hydrobiology, Chinese Academy of Sciences, Wuhan, China; ^2^ College of Advanced Agricultural Sciences, University of Chinese Academy of Sciences, Beijing, China

**Keywords:** starvation, cold stress, strain, endoplasmic reticulum stress, autophagy, apoptosis

## Abstract

Normally, fish will decrease food intake or even stop feeding during the winter. In previous studies, two widely cultured gibel carp strains (strain A and strain F) showed differences in lipid and glucose metabolism. Therefore, we hypothesized that the physiological changes during the overwintering period would be different between the two strains. Thus, the two strains were starved for 77 days, after which the levels of glucose and lipid metabolism, ER stress, autophagy, and apoptosis were determined. The starvation increased hepatic glycogenolysis and fatty acid β-oxidation but suppressed lipogenesis in both strains overwintering. Considering the effects of genotype, strain F had higher levels of ER stress and autophagy but lower levels of apoptosis than strain A, suggesting that strain F might be more resistant to overwintering starvation. The interactions between strains and starvation periods were observed in plasma triglyceride contents and the mRNA levels of pyruvate kinase (pk), sterol regulatory element binding protein 1 (srebp1), activating transcription factor 4 (atf4), and autophagy protein 12 (atg12). In conclusion, long-term starvation during winter could induce hepatic glycogenolysis and fatty acid β-oxidation but suppress lipogenesis, ER stress, autophagy, and apoptosis in gibel carp, and strain F may be more resistant to starvation during winter. Taken together, these results discovered the responses to prolonged starvation stress during winter in two strains of gibel carp and could provide information for genotype selection, especially for selecting strains better adapted to winter.

## Introduction

The fish breeding programs can improve economic efficiency through selection usually based on the growth performances of fish (1). However, stress resistance is also an important breeding goal in aquaculture. Determination of genotype differences in stressful environments will provide information for selecting strains better adapting various environments. Temperature is an important environmental factor that strongly affects growth, general behavior, reproductive performance, and immune responses of animals (2). During winter, homeothermic animals increase food intake to maintain body temperature, but poikilothermic animals such as fish will decrease food intake (3, 4) or even stop feeding (5). Effects of fasting on the chemical composition, morphological parameters, plasma metabolite contents, and activities and gene expression levels of key enzymes related to glucose and lipid metabolism have been extensively researched in fish (6–8). Overwintering fish may face stress from both starvation and cold. The genetics of overwintering performances in common carp (*Cyprinus carpio* and *Cyprinus rubrofuscus*) and overwintering energy mobilization in brook charr (*Salvelinus fontinalis*) have been reported (9, 10). However, the physiological responses to overwintering in different strains of fish are remain unclear. Chronic mortality of overwintering fish may be caused by disturbed physiology (11), and this has led to huge economic losses in aquaculture (12). Therefore, it is vital to investigate the physiological response to cold stress in fasting farmed fish especially in different strains of fish during winter.

It has been reported that various stress conditions, including nutrient deprivation, can lead to endoplasmic reticulum (ER) dysfunction followed by accumulation of misfolded proteins in the ER, which then invokes the unfolded protein response (UPR) (13, 14). The UPR helps to eliminate the misfolded proteins, thereby protecting cells from stress and maintaining cell homeostasis (15). The UPR is activated through three ER stress sensor proteins, inositol requiring enzyme 1 (IRE1), protein kinase RNA-like ER kinase (PERK), and activating transcription factor 6 (ATF6), that can sense stress conditions and then carry the information to the nucleus (16, 17). In addition, ER stressors can modulate autophagy during prolonged ER stress to play a vital role in cell survival (15, 18). Autophagy is a catabolic process in which the macromolecules and organelles in a cell are degraded via lysosomal action, thereby providing cells with energy by mobilizing cellular energy stores such as glucose and lipids during stressed conditions (19). Autophagosomal-lysosomal pathways play a vital role in the survival of fasting fish (20). For example, fasting helps to resist low temperature by regulating lipid catabolism and autophagy in zebrafish (4). A heightened degree/duration of ER stress can not only lead to the activation of autophagy but also cause to cell apoptosis (21). Apoptosis can be triggered by three main signaling pathways (the death receptor pathway, the mitochondrial pathway, and the ER pathway), upstream of caspase activation (22, 23). At present, the effects of fasting on ER stress, autophagy, and apoptosis of overwintering fish remain unclear.

Gibel carp (*Carassius gibelio*) is one of the most economically important species in fisheries and aquaculture of China, and its annual production of 2018 was about 3 million tons (24). The farmed gibel carp must cope with long-term cold and fasting during the winter. Two strains of gibel carp reproduced by unisexual gynogenesis, strain A (CAS III) and strain F (CAS V), showed differences in lipid metabolism and glucose metabolism in previous studies. The strain F exhibited better growth performance, lipid utilization, and glucose homeostasis ability than the strain A (25, 26). As a warm water fish, gibel carp stop moving and dramatically decrease feed intake or even stop feeding during winter. Therefore, we hypothesized that the physiological changes during the overwintering period would differ between the two strains. Thus, to investigate the physiological responses of the two gibel carp strains to starvation during winter, strain A and strain F were left unfed for 77 days, after which glucose and lipid metabolism, ER stress, autophagy, and apoptosis in the two strains were determined.

## Materials and Methods

### Fish and Trial Management

**Figure 1:**
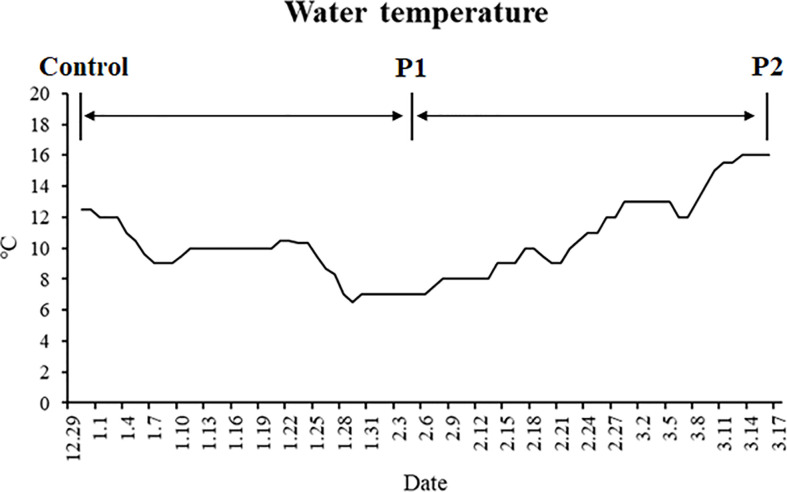
Changes in the water temperature during the experiment.

Two strains of gibel carp (strain A, body weight: 90.39 ± 0.33 g; strain F, body weight: 110.01 ± 0.19 g) were obtained from the hatchery of the Institute of Hydrobiology, the Chinese Academy of Sciences, Wuhan, Hubei, China. This experiment was performed according to the guiding principles for the care and use of laboratory animals and was approved by Institute of Hydrobiology, Chinese Academy of Sciences (Approval ID: IHB 2013724). Before the experiment, all experimental fish were reared in the tanks of a recirculation system for 2 weeks for acclimatization. During the 2 weeks, fish were fed with extruded feed twice a day. The experimental fish were then batch-weighed and randomly distributed into three fiberglass tanks for each strain (25 individuals per tank). After that, all the fish were starved for 77 days. The water temperatures during the trial are shown in **Figure 1**. Water quality was maintained as follows: dissolved oxygen > 6 mg L^−1^, total ammonia-nitrogen < 0.1 mg L^−1^, and residual chloride < 0.01 mg L^−1^.

### Sample Collection

Prior to the experiment, six fish from each strain were sampled at 6 h after the last feeding as a control. Then, the experimental fish were batch-weighed and two fish from each tank were sampled when the fish had fasted for 38 days (P1) and 77 days (P2). Fish were anaesthetized with 60 mg L^−1^ MS-222 (Sigma-Aldrich, St. Louis, MO, USA). Blood was quickly obtained from the caudal vein, and then, the plasma was separated after centrifugation for analysis. After that, the livers and muscles of fish were quickly sampled, and the tissue samples were stored at −80°C until analysis.

### Biochemical Analysis

Biochemical metabolites in plasma were determined using a biochemical analyzer (Mindray, Shenzhen, China). Glycogen contents were determined in livers and muscles (n = 6) using commercial kits (Liver/muscle glycogen kit, Jiancheng Bioengineering Institute, Nanjing, China). The activity of caspase 3 were determined in livers using commercial kits (Caspase 3 Activity Assay Kit, Beytime Biotechnology, Shanghai, China).

### Gene Expression Analysis

Total RNA of liver tissue was extracted using the TRIzol reagent (Invitrogen, Carlsbad, CA, USA) and then performed the DNAse treatment. The cDNA was obtained using M-MLV First-Strand Transcriptase (Invitrogen, Shanghai, China). Quantitative RT-PCR was performed on a LightCycler 480 System (Roche, Germany) with SYBR^®^ Green I Master Mix (Roche, Germany). EF1α was used as housekeeping gene, and PCR were performed using six biological replicates and two technical replicates along with negative controls without reverse transcriptase or template. Melting curves were monitored to confirm the specificity of the amplification reaction. Amplicon identities were confirmed by sequencing. **Table S1** shows the primers used for quantitative RT-PCR and amplification efficiencies. The calculation of relative quantification was performed using the Pfaffl’s mathematical model (27).

### Western Blot

Liver tissues were cell lysed by RIPA lysis buffer (Beyotime Biotechnology, China) containing protease inhibitor cocktail and phosphatase inhibitor cocktail (Roche, Basel, Switzerland). Proteins (40 μg) were separated on SDS-PAGE gels and then transferred to PVDF membranes. The membranes were blocked for 1 h by using 5% skimmed milk in TBST buffer (20 mM Tris-HCl, 150 mM sodium chloride, 0.1% Tween 20, pH 7.5), and then were incubated overnight at 4°C by using the following specific primary antibodies: BiP/GRP78 Antibody (1:1,000, #3177; Cell Signaling Technology, Danvers, MA, USA) or LC3A/B Antibody (1:1,000, #4108; Cell Signaling Technology, Danvers, MA, USA). After washing, membranes were incubated with secondary antibody: Goat Anti-Rabbit IgG H&L (HRP) (1:2000, ab205718; Abcam). The bands were acquired by ImageQuant LAS 4000mini (GE Healthcare Life Sciences) and quantified using Image J software (National Institutes of Health).

### Statistical Analysis

All data are presented as mean ± standard error (n = 6), and a two-way analysis of variance (ANOVA) was used to detect the signiﬁcance of differences between strains, periods, and interactions. The signiﬁcant difference level was considered *P* < 0.05.

## Results

### Body Weight Change

**Figure 2:**
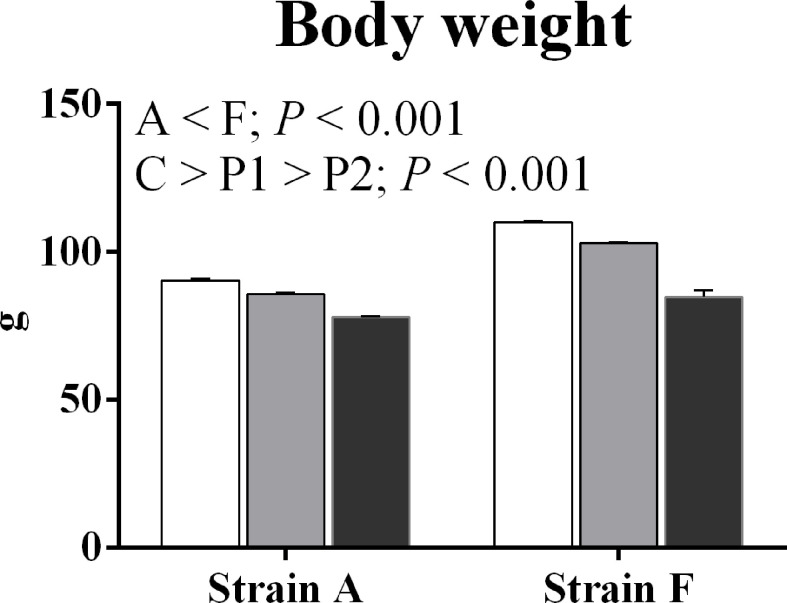
Body weight of two strains of gibel carp starved for 0 days (C, white bars), 38 days (P1, gray bars), and 77 days (P2, black bars) during winter.

The body weight of both two strains starved for 38 days (P1) and 77 days (P2) was significantly decreased compared with the initial body weight (*P* < 0.05) (**Figure 2**).

### Glucose Metabolism

**Figure 3:**
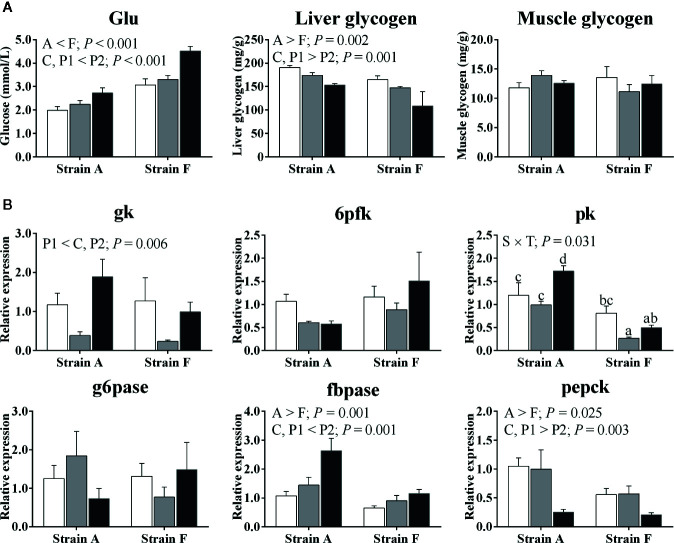
Plasma glucose levels, tissue glycogen contents **(A)** and expression of genes related to glucose metabolism in the livers **(B)** of two strains of gibel carp starved for 0 days (C, white bars), 38 days (P1, gray bars), and 77 days (P2, black bars) during winter. Data were presented as mean ± SE (n = 6), and the differences were evaluated by two-way ANOVA (*P* < 0.05). When interactions were identified, different lowercase letters represented the significant differences among all groups (*P* < 0.05). Glu, glucose; gk, glucokinase; g6pase, glucose-6-phosphatase; 6pfk, 6-phosphofructokinase; fbpase, fructose-1,6-bisphosphatase; pk, pyruvate kinase; pepck, phosphoenolpyruvate carboxykinase.

Plasma glucose levels increased in both strains (*P* < 0.05) (**Figure 3A**). Strain F had higher plasma glucose levels than strain A (*P* < 0.05). Liver glycogen contents decreased in both strains (*P* < 0.05), and strain A had higher contents than strain F (*P* < 0.05). No significant changes were found in muscle glycogen contents (*P* > 0.05).

The expression levels of genes involved in glucose metabolism are shown in **Figure 3B**. Regarding glycolysis, both strains exhibited a significant decrease in the mRNA levels of glucokinase (gk) at P1. A significant decrease in the mRNA levels of pyruvate kinase (pk) was only found in strain F (*P* < 0.05), while in strain A, expression levels of pk were only increased at P2 (*P* < 0.05). There were no significant differences in gene expression levels of 6-phosphofructokinase (6pfk) between strains or time points (*P* > 0.05). No changes were detected in mRNA levels of glucose-6-phosphatase (g6pase) irrespective of strains or time points (*P* > 0.05). The gene expression levels of fructose-1,6-bisphosphatase (fbpase) were significantly increased in both strains at P2 (*P* < 0.05), while gene expression levels of phosphoenolpyruvate carboxykinase (pepck) were significantly decreased at P2 (*P* < 0.05). Higher fbpase and pepck expression levels were found in strain A compared to strain F (*P* < 0.05).

### Lipid Metabolism

**Figure 4:**
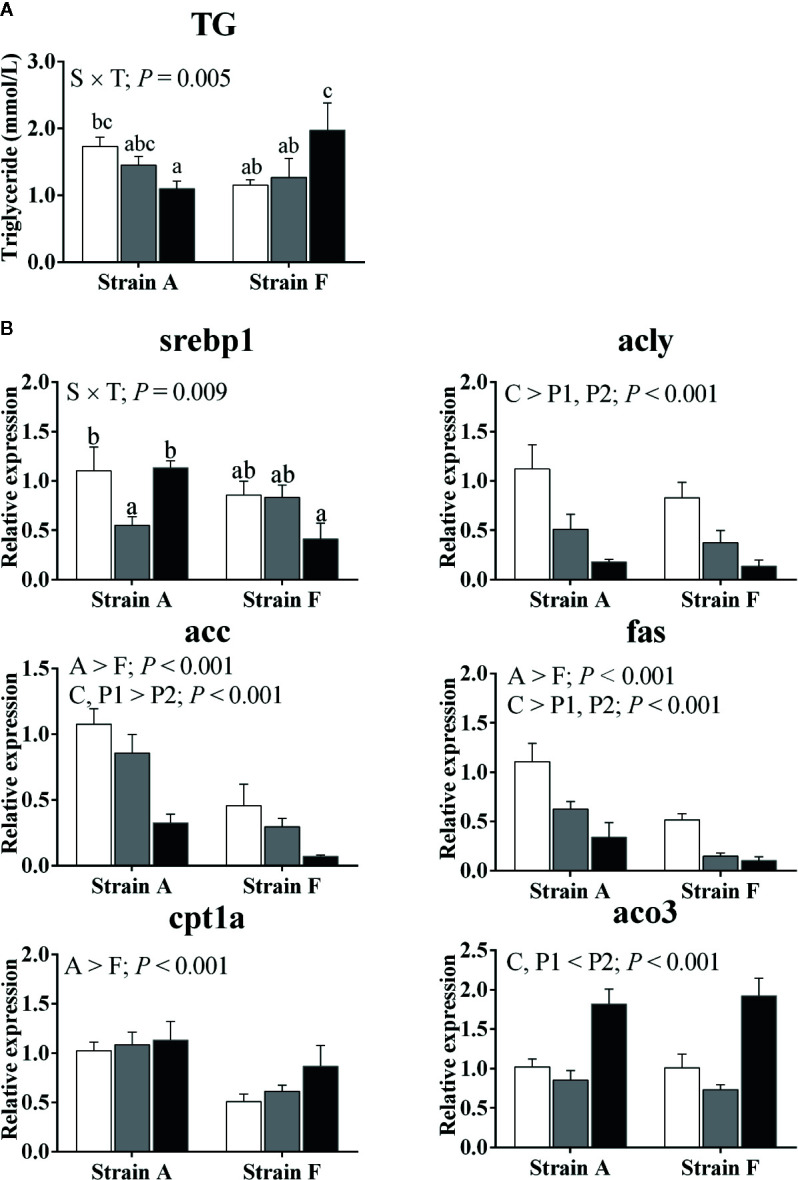
Plasma triacylglycerol levels **(A)** and expression of genes related to lipid metabolism in the livers **(B)** of two strains of gibel carp starved for 0 days (C, white bars), 38 days (P1, gray bars), and 77 days (P2, black bars) during winter. Data are presented as mean ± SE (n = 6), and the differences were evaluated by two-way ANOVA (*P* < 0.05). When interactions were identified, different lowercase letters represented the significant differences among all groups (*P* < 0.05). TG, triacylglycerol; srebp1, sterol regulatory element binding protein 1; acly, ATP citrate lyase; acc, acetyl-CoA carboxylase; fas, fatty acid synthase; cpt1a, carnitine palmitoyl transferase 1 isoform a; aco3, acyl-CoA oxidase 3.

An interaction was found between strain and starvation period in plasma triglyceride levels; this decreased afterward in strain A (*P* < 0.05), while increasing in strain F (*P* < 0.05) (**Figure 4A**).

Gene expression levels of key enzymes involved in lipid metabolism are presented in **Figure 4B**. An interaction between strain and time was found in the mRNA levels of sterol regulatory element binding protein 1 (srebp1), which was only decreased in strain A at P1 (*P* < 0.05), while there was no corresponding significant change in strain F. The transcript levels of ATP citrate lyase (acly) and fatty acid synthase (fas) were significantly decreased at P1 and P2 (*P* < 0.05). Acetyl-CoA carboxylase (acc) transcript levels were decreased in both strains only at P2 (*P* < 0.05), while the inverse change was found in acyl-CoA oxidase 3 (aco3) (*P* < 0.05). The expression levels of carnitine palmitoyl transferase 1 isoform a (cpt1a) were higher in strain A than in strain F (*P* < 0.05), and the mRNA levels were significantly increased in both strains only at P2 (*P* < 0.05).

### ER Stress, Autophagy, and Apoptosis

**Figure 5:**
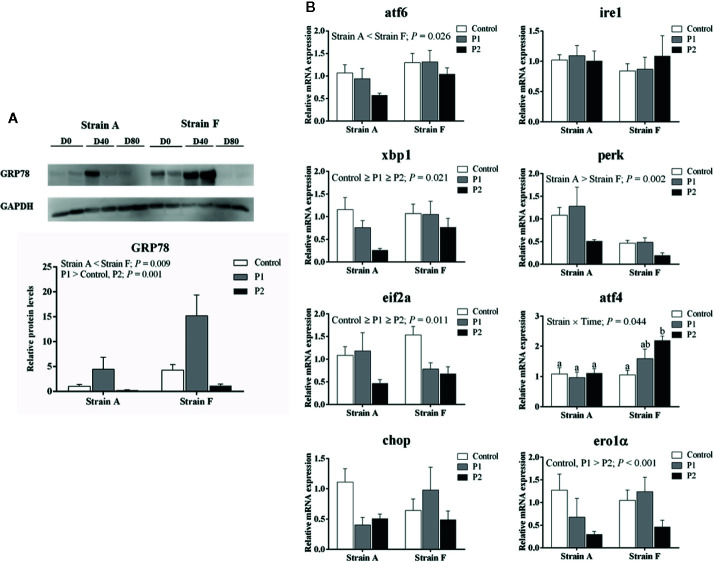
Protein levels of GRP78 **(A)** and expression levels of genes related to ER stress **(B)** in the livers of two strains of gibel carp starved for 0 days (C, white bars), 38 days (P1, gray bars), and 77 days (P2, black bars) during winter. Data were presented as mean ± SE (n = 6), and the differences were evaluated by two-way ANOVA (*P* < 0.05). When interactions were identified, different lowercase letters represented the significant differences among all groups (*P* < 0.05). GRP78, 78-kDa glucose-regulated protein; atf6, activating transcription factor 6; ire1, inositol-requiring protein-1α; xbp1, X-box-binding protein 1; perk, eukaryotic translation initiation factor 2-alpha kinase 3; eif2a, eukaryotic translation initiation factor 2A; atf4, activating transcription factor 4; chop, DNA damage-inducible transcript 3 protein; ero1α, endoplasmic reticulum oxidoreductase 1 alpha.

We analyzed the protein levels of 78-kDa glucose-regulated protein (GRP78) and LC3B (Microtubule-associated protein 1 light chain-3B) and the expression levels of genes involved in ER stress. The protein levels of GRP78 were increased at P1 (*P* < 0.05), then decreased at P2 in both strains (*P* < 0.05), and strain F showed higher levels than strain A, irrespective of time points (*P* < 0.05) (**Figure 5A**). As shown in **Figure 5B**, the mRNA levels of X-box-binding protein 1 (xbp1), eukaryotic translation initiation factor 2A (eif2a) and ER oxidoreductase 1 alpha (ero1α) were significantly decreased at P2 in both strains, but there were no changes in the levels of atf6, perk, ire1 or DNA damage-inducible transcript 3 protein (chop) related to starvation period (*P* > 0.05). Strain F showed the higher expression level of activating transcription factor 4 (atf4) at P2, but no differences were observed in strain A. Strain A showed lower levels of atf6 and higher levels of perk than strain F.

**Figure 6:**
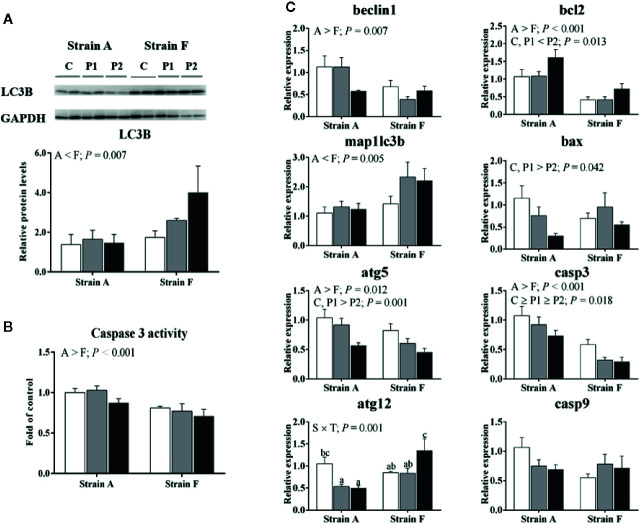
Protein levels of LC3B **(A)**, caspase 3 activity **(B)** and expression levels of genes related to autophagy and apoptosis **(C)** in the livers of two strains of gibel carp starved for 0 days (C, white bars), 38 days (P1, gray bars), and 77 days (P2, black bars) during winter. Data are presented as mean ± SE (n = 6), and the differences were evaluated by two-way ANOVA (*P* < 0.05). When interactions were identified, different lowercase letters represented the significant differences among all groups (*P* < 0.05). LC3B, Microtubule-associated protein 1 light chain-3B; map1lc3b, microtubule-associated proteins 1A/1B light chain 3B; atg5, autophagy protein 5; atg12, autophagy protein 12; bcl2, apoptosis regulator Bcl-2; bax, BCL2 associated X, apoptosis regulator; casp3, caspase 3; casp9, caspase 9.

We analyzed the protein levels of LC3B (Microtubule-associated protein 1 light chain-3B). As presented in **Figure 6A**, strain F showed higher protein levels of LC3B than that of strain A (*P* < 0.05). The activities of caspase 3 were significantly higher in strain A than that in strain F (*P* < 0.05) (**Figure 6B**). The expression levels of genes involved in autophagy and apoptosis are shown in **Figure 6C**. No differences were found in gene expression levels of beclin1 or microtubule-associated proteins 1A/1B light chain 3B (map1lc3b), regardless of time point (*P* > 0.05). The mRNA levels of map1lc3b in strain F was significantly higher compared to strain A (*P* < 0.05). The mRNA levels of autophagy protein 5 (atg5) were decreased at P2 in both strains (*P* < 0.05), and autophagy protein 12 (atg12) levels were decreased at P1 in strain A but increased at P2 in strain F (*P* < 0.05). Strain A had higher levels of beclin1 and atg5 than strain F (*P* < 0.05). Higher apoptosis regulator Bcl-2 (bcl2) and lower bcl2 associated X, apoptosis regulator (bax), and caspase 3 (casp3) mRNA levels were found in both fish at P2 (*P* < 0.05). Unaltered mRNA levels of caspase 9 (casp9) were found irrespective of strain and time point (*P* > 0.05). Strain A exhibited higher mRNA levels of bcl2 and casp3 than strain F (*P* < 0.05).

## Discussion

### Physiological Responses to Overwinter Starvation

Significantly increased plasma glucose levels were observed in fish starved for 77 days during winter in the present study. The different changes in plasma glucose levels might be related to the different durations of fasting, as suggested by the results of a study on gilthead sea bream (*Sparus aurata*) (5). In the present study, glycogenolysis in the liver might be activated to provide energy under starvation, as indicated by the liver glycogen content being decreased in fish starved for 77 days (8).

Glucokinase (gk) is the first key enzyme involved in glycolysis, and it has been shown that liver gk mRNA levels were suppressed in 14-day starved rainbow trout (28). In the present study, the mRNA abundance of gk in the liver was dramatically decreased at P1, but mRNA levels of gk were surprisingly increased at P2, which might be associated with the duration of starvation (29). It has been reported that gluconeogenesis genes (pepck, fbpase, and g6pase) can be induced in starved fish (8, 30). Indeed, an increase in the expression of fbpase was observed in fish starved for 77 days. However, g6pase mRNA levels showed no changes in the present study, which was similar to the study in Siberian sturgeon (*Acipenser baerii*) (31). In addition, our data showed that lower pepck mRNA levels were found in fish starved for 77 days, in line with the results in Siberian sturgeon starved for 21 days (31). It seems that glucose metabolism can be influenced by fasting during winter.

In the present results, srebp1 (a key regulator of lipid biosynthetic genes) and acly, acc, and fas, which are involved in lipogenesis, exhibited lower mRNA levels in starved fish. Similar results were also observed in rainbow trout, gibel carp, and zebrafish (4, 8, 32). Furthermore, our data showed that fatty acid oxidation was enhanced, as reflected by higher aco3 mRNA levels in fish starved for 77 days. It has been suggested that during starvation, fatty acids would be mobilized as a source of energy in fish (4, 8). Thus, our data confirmed that starvation and low temperature could mobilize lipids as energy sources by suppressing lipogenesis and inducing fatty acid β-oxidation.

Nutrient deficiencies and low temperature have been reported to cause ER stress (33, 34). GRP78 is an important protective molecular chaperone which involved in stress-induced autophagy regulation (17). In our results, GRP78 protein levels were dramatically increased at P1, indicating that ER stress was induced. Moreover, the expression of ER-related genes was decreased in zebrafish starved for 72 h under cold stress, indicating that starvation could be a protective strategy for fish to survive low temperatures (4). However, lower GRP78 protein levels were observed in the fish at P2, where there was no difference with that of the control group, suggesting that ER stress was alleviated at P2. Accordingly, our data showed that the mRNA levels of xbp1 and eif1a were significantly decreased in fish starved for 77 days, whereas there were no changes in mRNA levels of atf6, perk, or ire1. Generally, the UPR is activated through the three ER stress sensor proteins ire1, perk and atf6 (17). Ire1 dimerizes and splices the mRNA of xbp1, and perk is activated to phosphorylate eif2a, which helps to recover ER homeostasis during ER stress (35–37). Furthermore, the mRNA levels of chop also showed no changes in the present study. Chop could be increased by ire1, perk and atf6-dependent transcriptional induction in response to ER stress, and the activation of atf4 induced by eif2a showed a dominant effect on the induction of chop (38). Although chop mRNA levels were stable, fish starved for 77 days had lower mRNA levels of ero1α, which is targeted by chop to hyperoxidize the lumen of the ER (39). Thus, our data suggested that the ER stress might have been induced at P1, but ER homeostasis may have recovered at P2.

Autophagy is regarded as a vital protein degradation pathway during starvation or stress (19), and it plays a significant role in cell survival after ER stress (18). Our data showed significantly reduced mRNA levels of atg5 in fish at P2. The autophagy-related 12 (atg12)-atg5 and microtubule-associated protein 1 light chain 3 (map1lc3, namely lc3) phosphatidylethanolamine conjugation systems are necessary in the expansion stage of autophagosome formation (19). It has been reported that 72 h starvation can induce autophagy in zebrafish under low temperature (4), but this was not found in our data, possibly related to the long-term starvation in our study. Therefore, our data indicated that the autophagy would be suppressed in fish at P2.

It has been reported that starvation can induce apoptosis, and that prolonged ER stress might result in apoptosis (40, 41). Bcl2 can promote cell proliferation and suppress apoptosis, and bax can accelerate cell apoptosis (42). In the present study, the mRNA levels of bcl2 were increased in fish at P2, while bax mRNA levels showed no changes. However, feed restriction was reported to alleviate transcription and translation levels of bcl2 but to enhance that of bax in granulosa cells (43). The mRNA levels of bax were significantly induced in myocardium of mice under 2-week cold exposure, while expression of mRNA in bcl2 decreased (44). Considering that chop was reported as a multifunctional transcription factor involved in ER stress-induced apoptosis (45), regulation of bcl2 and bax is considered to contribute to the apoptosis mediated by chop (46, 47). It has been suggested that the unaltered mRNA levels of bax might be related to the unchanged expression levels of chop. The induced mRNA levels of bcl2 and decreased mRNA levels of casp3 indicated that apoptosis might be suppressed in fish starved for 77 days, in line with the alleviated ER stress at P2 as discussed above. Compared to the water temperature in the natural situation, the temperature in experimental system was a bit higher, though the temperature trend was quite similar (48). Thus, physiological responses of fish induced by starvation might be stronger in outdoor aquaculture.

### Different Physiological Responses to Fasting in Overwintering Strains

Strain A had lower plasma glucose levels but higher liver glycogen contents and higher mRNA levels of fbpase and pepck than strain F. The previous results showed that these two strains had no significant differences in mRNA of fbpase or pepck when fed different diets (25, 26). Therefore, we suggested that strain A and strain F might have different responses in glucose metabolism to starvation and low temperature. As gluconeogenesis can be induced by starvation (8, 30), this indicated that strain A would have a stronger response in gluconeogenesis than strain F. These two strains have been reported to have no significant differences in mRNA levels of acc, fas, or cpt1a when fed diets containing different lipid or glucose levels, but there were differences when the fish were fed different protein sources (25, 26, 49). Moreover, our data showed that strain A exhibited higher mRNA levels of acc, fas, and cpt1a than strain F. Fasting would induce fatty acid oxidation but decrease lipogenesis in fish (4, 8), suggesting that strain A exhibited higher levels of lipid metabolism to cope with the overwintering starvation compared to strain F.

In the present study, strain A had lower protein levels of GRP78 than strain F, which indicated that strain F might have a better ability to alleviate the stress of fasting over winter. Our data showed that strain A had lower mRNA levels of atf6 but higher mRNA levels of perk than strain F. Thus, it seemed that ER stress was mainly mediated through atf6 rather than perk and ire1 in strain F compared to strain A. In the present study, higher gene expression levels of atg5 but lower mRNA levels of map1lc3b were observed in strain A compared to strain F. Considering that atg5 and map1lc3b are involved in the expansion stage of autophagosome formation (19), this result indicated that strain A and strain F showed different responses to the starvation during winter regarding autophagosome expansion. Beclin1 has been reported to induce autophagy (50). In the present study, the mRNA levels of beclin1 were higher in strain A than in strain F. Actually, the regulation of beclin 1 on autophagy was affected by post-translational modifications (51). Strain F showed higher protein levels of LC3B than that of strain A, indicating that higher levels of autophagy in strain F than in strain A, in lines with previous results (52). Our data showed that strain A exhibited higher mRNA levels of bcl2 and casp3. Similarly, caspase 3 activities were also higher in strain A. These results indicated that stronger cell apoptosis was induced in strain A, in agreement with results in the previous study (52). It has been reported that fasting and low temperature could induce apoptosis in fish (41). Thus, strain A exhibited higher expression levels of genes related to apoptosis, and thus might be more susceptible to the effects of starvation during winter. Therefore, the results suggested that the two strains showed different responses in autophagy and apoptosis to the fasting during winter, and strain F had higher levels of autophagy but lower levels of apoptosis than strain A.

### Interactions Between Strain and Starvation Period

An interaction for pk mRNA levels between genotypes and starvation periods was found in the present study. It has been reported that the mRNA levels of pk were not affected by starvation, except in strain A of gibel carp starved for 21 days (8). Indeed, higher levels of pk mRNA were observed in stain A starved for 77 days. However, strain F starved for 38 and 77 days exhibited lower expression levels. Previous results showed that strain A had higher gene expression levels of pk than strain F when the fish were fed diets containing different levels of carbohydrate or lipid (25, 26). In the present study, strain A still presented higher pk mRNA levels than strain F under starvation during winter. Regarding lipid metabolism, interactions were found in the plasma triglyceride contents and srebp1 mRNA levels. Our data showed that plasma triglyceride contents of strain A decreased over time, while those in strain F increased. Triglycerides are considered as the most accessible form of lipid storage during lipolysis induced by starvation (53). Moreover, a study in trout indicated that fish exported lipids from the liver as lipoproteins to adapt to the low temperature, and that this might cause persistent hypertriglyceridemia (54). The lowest mRNA levels of srebp1 were found in strain A starved for 38 days but in strain F starved for 77 days. It was obvious that the change trends of srebp1 mRNA levels in strain F were in line with those of lipogenesis genes. Thus, we speculated that the lipid metabolism of strain A might have been disrupted during the starvation period, as was also reported in the gilthead sea bream (12). The mRNA levels of atf4 and atg12, which are involved in ER-stress and autophagy, showed significant interaction between strains and starvation period. The expression levels of atf4 exhibited no change in strain A but showed higher levels in strain F starved for 77 days. The induction of PERK would suppress general protein translation, and then the open reading frame ATF4 would be encoded to activate downstream UPR target genes (36, 55). Although strain F had lower expression levels of perk, it seems that the downstream genes could be induced in strain F to maintain cellular environmental homeostasis. Atf4 was reported to induce autophagy by regulating mRNA levels of atg genes (56). Indeed, the mRNA levels of atg12 were decreased in strain A at P1 and P2 but were increased in strain F at P2, in line with the expression levels of atf4.

## Conclusions

In summary, we found that long-term starvation during winter could induce hepatic glycogenesis and fatty acid β-oxidation but suppress lipogenesis, ER stress, autophagy, and apoptosis in both strains of gibel carp. Moreover, strain F had higher levels of ER stress and autophagy but lower levels of apoptosis than strain A, possibly indicating that strain F may be more resistant to starvation during winter. The present study can provide information for exploring physiological responses of overwintering fish and for selecting new strains better adapted to winter.

## Data Availability Statement

The datasets presented in this study can be found in online repositories. The names of the repository/repositories and accession number(s) can be found in the article/**Supplementary Material**.

## Ethics Statement

The animal study was reviewed and approved by the Institute of Hydrobiology, Chinese Academy of Sciences. Written informed consent was obtained from the owners for the participation of their animals in this study.

## Author Contributions

JJ designed the experiment and revised the manuscript. WX performed the experiment and drafted the manuscript. HLi, LW, and BD performed the statistical analysis. YY contributed to the sample analysis. DH, XZ, HLiu, and SX provided suggestions on the experimental design and contributed to the manuscript modification. All authors contributed to the article and approved the submitted version.

## Funding

This work was financially supported by the National Natural Science Foundation of China (U19A2041; 31972805), the National Key R&D Program of China (2018YFD0900605; 2019YFD0900200), the China Agriculture Research System (CARS-45-09), and the Science and Technology Service Network Initiative (Y82Z131201).

## Conflict of Interest

The authors declare that the research was conducted in the absence of any commercial or financial relationships that could be construed as a potential conflict of interest.
