# Supplementary material for: Genetically Based Physiological Responses to Overwinter Starvation in Gibel Carp (Carassius gibelio)
Source: Front Endocrinol (Lausanne). 2020 Nov 19;11:578777. doi: 10.3389/fendo.2020.578777 (PMC7711150; doi:10.3389/fendo.2020.578777)
Supplement: Supplementary file 1 [file Table_1.docx]

**Table S1 Sequences of the primers used for qRT-PCR analysis in gibel carp.**

| Gene | Acronym | Prime sequence | Amplicon size (bp) | Amplification efficiency | Accession No. |
| --- | --- | --- | --- | --- | --- |
| Elongation factor 1 alpha | ef1α | GTTGGAGTCAACAAGATGGACTCCAC | 198 | 2.03 | AB056104 |
|  |  | CTTCCATCCCTTGAACCAGCCCAT |  |  |  |
| Glucokinase | gk | GAGGAGATGCGTAAGGTGGAGCT | 167 | 1.99 | KX898498 |
|  |  | TTCTCATACAGCTGATGTCCAGGGTT |  |  |  |
| Pyruvate kinase | pk | GCATCTGTGTCTGCTGGACATCGA | 144 | 1.96 | KX898502 |
|  |  | TGAGAGCCGTGAGAGAAGTTCAGTC |  |  |  |
| 6-phosphofructokinase | 6pfk | ACACCGGATGCCGCAGAAGCA | 105 | 1.96 | KX898500 |
|  |  | TCGATCTCTCCGGTCACATACTCG |  |  |  |
| Glucose-6-phosphatase | g6pase | CCTTACTGGTGGGTCCATGAGACT | 90 | 1.98 | KX898505 |
|  |  | TGGGCCGGTCTCACAGGTCAT |  |  |  |
| Fructose-1,6-bisphosphatase | fbpase | CACAAATGTTACAGGTGACCAGGTGAA | 193 | 2.01 | GU593002 |
|  |  | AATGTTTGAAGAGCCATCCAGAGGGT |  |  |  |
| Phosphoenolpyruvate carboxykinase | pepck | AGACAAACCCTCATGCCATGGCAAC | 226 | 1.95 | KX898506 |
|  |  | GGGTCTATGATGGGGCACTGG |  |  |  |
| Fatty acid synthase | fas | CCACACCATGGACCCACAGCT | 158 | 2.05 | KF511494 |
|  |  | CTGGGTCTTTACTGAAGGCCTCT |  |  |  |
| Sterol regulatory element binding protein 1 | srebp1 | GGCCCTCTACTGCGTGGCACA | 194 | 2.09 | KX898507 |
|  |  | ACCACCATTTGGAGTGAGGGTCAC |  |  |  |
| ATP citrate lyase | acly | AGTTTGGCCACGCTGGAGCTTGT | 112 | 2.03 | KX898508 |
|  |  | CCCAGCTCATCGAAGCTCTTGG |  |  |  |
| Acetyl-CoA carboxylase | acc | GAGCTGTCTATCAGAGGAGACTTCA | 139 | 1.99 | KF499584 |
|  |  | GACGCTCGGCCTGCATCTTCT |  |  |  |
| Carnitine palmitoyl transferase 1 isoform a | cpt1a | GAAGCTCATCAGGCTGTGGCCTT | 113 | 2 | KX898509 |
|  |  | TTCCAGGAGTGAAGTCCGGAGAG |  |  |  |
| Acyl-CoA oxidase 3 | aco3 | TGTGGAGGACACGGTTACCTTGC | 115 | 1.99 | KX898510 |
|  |  | AGTTGCTGGTCTGCTGCAGAAGG |  |  |  |
| Activating transcription factor 6 | atf6 | TGCAGGTGTATTACGCCCCTCAC | 176 | 2.01 | XM_026290872.1 |
|  |  | GTAATTCATAGCTGGCAGGACCAC |  |  |  |
| Eukaryotic translation initiation factor 2A | eif2a | AGCTGCCAAAGAACGGCCCCATT | 226 | 1.99 | XM_026230526.1 |
|  |  | CAAACTTCCATCTGCCCTCTCAG |  |  |  |
| Inositol-requiring protein-1α | ire1 | GCGACCTTTCCTGCCTTACT | 253 | 1.98 | XM_026218282.1 |
|  |  | AGTCTCCTGTTTGGACAGCG |  |  |  |
| X-box-binding protein 1 | xbp1 | CATCTACACCAAACCCACCGA | 264 | 2.05 | MN852578 |
|  |  | CATCCAGAGTCACTGTACGCA |  |  |  |
| Eukaryotic translation initiation factor 2-alpha kinase 3 | perk | TGCCATCAAGAGGATCCGTCTGC | 122 | 1.99 | XM_026224076.1 |
|  |  | CCTGCCAAGCATTGAAGTAACGG |  |  |  |
| Activating transcription factor 4 | atf4 | CAGCCGAGAGATCCGCTATC | 215 | 1.99 | XM_026260813.1 |
|  |  | GATGAGCCCCTTACTGGACG |  |  |  |
| DNA damage-inducible transcript 3 protein | chop | ACCACTCCTCGCTGACAGA | 88 | 1.98 | XM_026265784.1 |
|  |  | TTAGAGGCCTCGGGTCGAT |  |  |  |
| Endoplasmic reticulum oxidoreductase 1 alpha | ero1α | ATGCCCAACACAAGCAACAC | 129 | 1.98 | XM_026242578.1 |
|  |  | TGACAACAGCGACCGAAAGT |  |  |  |
| Microtubule-associated proteins 1A/1B light chain 3B | map1lc3b | CTACGAGCGCGAGAGAGATG | 81 | 2.02 | XM_026238789.1 |
|  |  | TGAGGACACGCAGTTCCAAA |  |  |  |
| Beclin-1 | beclin1 | TGGAGAACTTGAGTCGCAGG | 129 | 2.02 | XM_026249455.1 |
|  |  | GCTGAGTGTCCAGATGGTCG |  |  |  |
| Autophagy protein 5 | atg5 | GCTCTTCCGACCAGTGTCTC | 188 | 2 | XM_026284696.1 |
|  |  | AGTTGTCTGGGTGGCTCAAG |  |  |  |
| Autophagy protein 12 | atg12 | GCTGTTGAAAGCAGTAGGTGATG | 170 | 1.98 | XM_026284438.1 |
|  |  | GGTCTGGTGATGGAGCAAATGAC |  |  |  |
| Apoptosis regulator Bcl-2 | bcl2 | AAAGGATGTACCAGCGCGAA | 83 | 2.03 | XM_026237836.1 |
|  |  | GGCTAAGAATCTGCGTTGCG |  |  |  |
| BCL2 associated X, apoptosis regulator | bax | ACCCCAGCCATAAACGTCTTGCG | 214 | 2.02 | XM_026262399.1 |
|  |  | GCCTTGATGACAAGCCGACAC |  |  |  |
| Caspase 3 | casp3 | ATCATGACCAGGGTCAACCA | 119 | 2.01 | XM_026266756.1 |
|  |  | TACATCTCTTTGGTGAGCAT |  |  |  |
| Caspase 9 | casp9 | ATCACAAACTACCTCAACGG | 80 | 1.99 | XM_026241892.1 |
|  |  | CCTCCACAGGCCTGGATGAA |  |  |  |
